# Neuroprotective effects of magnesium: implications for neuroinflammation and cognitive decline

**DOI:** 10.3389/fendo.2024.1406455

**Published:** 2024-09-25

**Authors:** Veer Patel, Nuraly S. Akimbekov, William B. Grant, Carolyn Dean, Xiaoqian Fang, Mohammed S. Razzaque

**Affiliations:** ^1^ Department of Pathology, Lake Erie College of Osteopathic Medicine, Erie, PA, United States; ^2^ Scientific-Practical Center, West Kazakhstan Marat Ospanov Medical University, Aktobe, Kazakhstan; ^3^ Sustainability of Ecology and Bioresources, Al-Farabi Kazakh National University, Almaty, Kazakhstan; ^4^ Sunlight, Nutrition, and Health Research Center, San Francisco, CA, United States; ^5^ New Capstone, Inc., Mooresville, NC, United States; ^6^ Department of Neuroscience, School of Medicine, University of Texas Rio Grande Valley (UTRGV), Edinburg, TX, United States; ^7^ Department of Medical Education, School of Medicine, University of Texas Rio Grande Valley (UTRGV), Edinburg, TX, United States

**Keywords:** magnesium, neuroinflammation, neurodegenerative disease, cognitive decline, neuroprotection

## Abstract

Neurodegenerative diseases, which are characterized by progressive neuronal loss and cognitive decline, are a significant concern for the aging population. Neuroinflammation, a shared characteristic of these diseases, is implicated in their pathogenesis. This article briefly summarizes the role of magnesium, an essential mineral involved in numerous enzymatic reactions and critical for neuronal bioactivity, in the context of neuroinflammation and cognitive decline. The potential neuroprotective effects of magnesium, including the mechanisms of neuroprotection by magnesium through maintaining neuronal ion homeostasis, reducing inflammation, and preventing excitotoxicity, are also described. Additionally, we discuss the impact of inadequate magnesium on neuroinflammation and its potential as a therapeutic agent for attenuating cognitive decline to improve neurodegenerative conditions.

## Introduction

As the global population ages, neurodegenerative diseases, which are characterized by an ongoing loss of neuron structure and function, are becoming increasingly public health burdens. These disorders, including dementia (along with vascular dementia), amyotrophic lateral sclerosis (ALS), Alzheimer’s disease, Parkinson’s disease, and Huntington’s disease, often result in cognitive decline, which severely impacts the quality of life of affected individuals. The insidious nature of these diseases and the lack of curative interventions highlight the need for novel therapeutic strategies. The global prevalence of dementia, primarily Alzheimer’s disease, is expected to double every 20 years, reaching 81.1 million by 2040 (1, 2). Similarly, Parkinson’s disease, the second most common neurodegenerative disorder, affects 2-3% of the population aged ≥65 years (3, 4). These statistics highlight the escalating public health challenge of neurodegenerative diseases.

**Figure 1:**
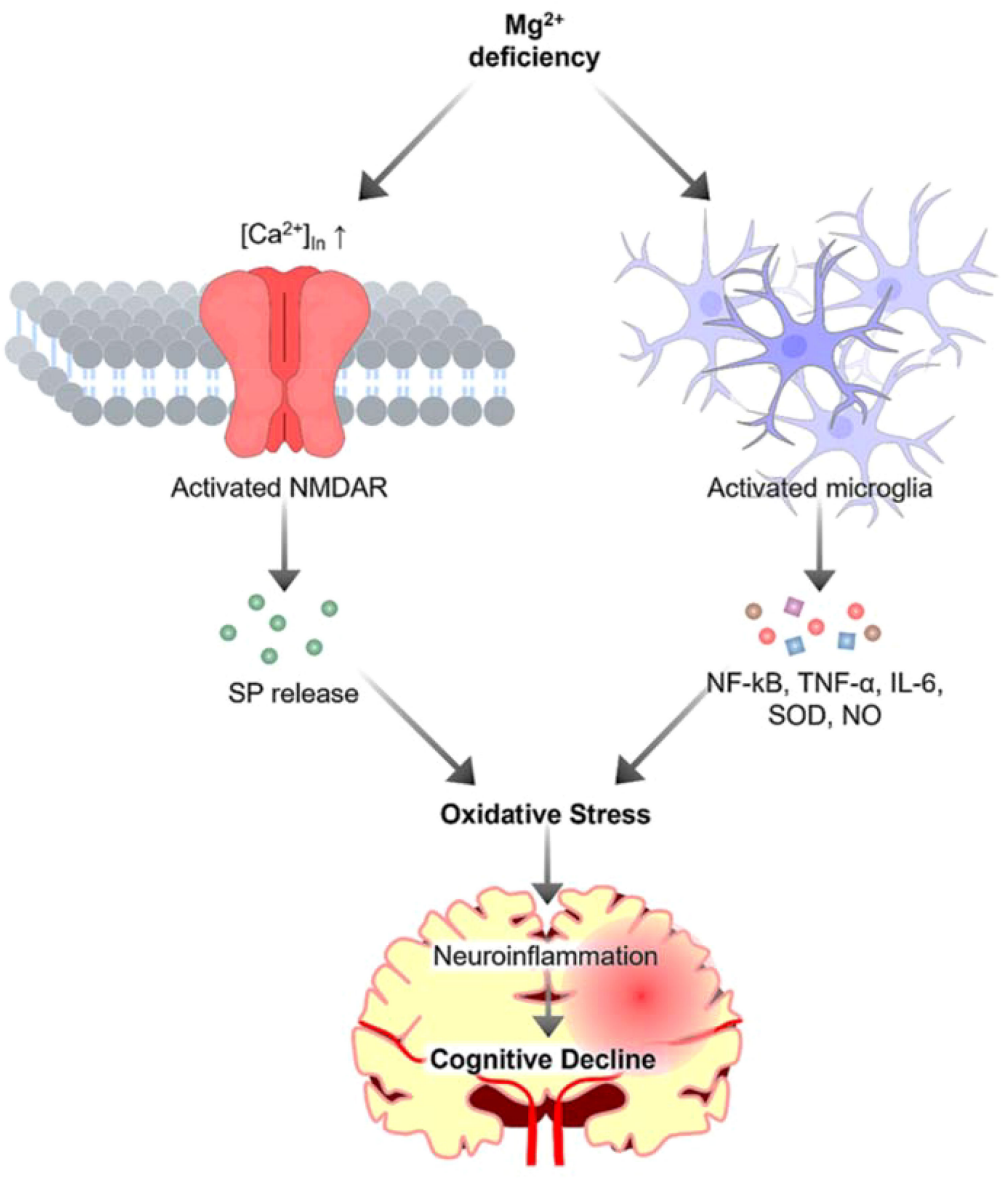
The role of magnesium in neuroinflammation. Magnesium deficiency activates microglia, resulting in the release of proinflammatory cytokines and toxic substances, which contribute to oxidative stress. Additionally, magnesium deficiency triggers calcium influx, inducing the release of substance P (SP), further exacerbating oxidative stress to increase neuroinflammation and ultimately contributes to cognitive decline.

Neuroinflammation, a common feature of neurodegenerative diseases, is recognized as a critical player in the pathogenesis of these disorders (5, 6). The inflammatory response in the brain is a double-edged sword. Whereas acute inflammation can be beneficial for neuronal repair and recovery, chronic inflammation can lead to persistent neuronal damage and eventually to neurodegeneration (7). Inflammatory processes in the brain are primarily mediated by microglia shown in **Figure 1**. Upon activation, microglia release proinflammatory cytokines, including interleukin 1β (IL-1β), tumor necrosis factor -α (TNF-α), IL-6, IL-18, and IL-12, reactive oxygen species (ROS), and other neurotoxic substances, including nitric oxide, glutamate, and prostaglandins, as well as enzymes such as matrix metalloproteinases (MMPs) (7). Given the critical role of neuroinflammation in neurodegeneration, modulating the inflammatory response could reduce disease progression and is likely to improve clinical outcomes.

We conducted a literature search using PubMed, Google Scholar, and Scopus databases. The search was performed using the keywords: “neuroinflammation”, “magnesium”, “cognitive function” and “neurodegenerative diseases”. We included peer-reviewed articles in English published between 2000 and 2023.

## Neuroinflammation and neurodegeneration

Neuroinflammation is partly mediated by the activation of glial cells and the release of proinflammatory mediators in the brain (8). It plays a crucial role in the pathogenesis of neurodegenerative diseases. Magnesium has been shown to modulate neuroinflammation (9, 10). It is recognized for its diverse roles in maintaining human health, specifically in modulating inflammatory signaling pathways within the neurological landscape. Magnesium plays a crucial role in over 600 enzymatic reactions in the human body (11). According to Workinger, “Magnesium is a critical mineral in the human body and is involved in ~80% of known metabolic functions” (12). The concentrations of magnesium in serum and cerebrospinal fluid (CSF) are regulated to maintain normal physiological function. Normal serum magnesium levels typically range from 0.75 to 0.95 mmol/L (13), while in CSF, they range between 0.77 and 1.17 mmol/L (14). Magnesium levels are generally higher in CSF as compared to the serum levels, perhaps due to the active transport of magnesium across the blood-brain barrier (15); the blood-brain barrier and the choroid plexus help regulate magnesium levels in the CSF. In magnesium deficiency state, CSF concentrations decline, although such reduction lags behind and is usually less pronounced than the changes noted in plasma levels of magnesium (15). Serum magnesium levels are crucial for neuromuscular function, enzyme activity, and bone structure (16). Magnesium in CSF plays a vital role in supporting various functions of the central nervous system. Decreased CSF magnesium levels correspond with reduced concentrations of extracellular brain magnesium and have been associated with epilepsy (14). Additionally, magnesium is well known for its implication in multiple neurological disorders (17). For instance, magnesium sulfate supplementation has been associated with reduced neuroinflammation in a rat model of Alzheimer’s disease (10). Studies involving animal models suggest that magnesium deficiency may trigger greater recruitment of phagocytic cells (18). These cells could lead to generation of more ROS, leading to the production of various cytokines, such as TNF-α, which are key players in the inflammatory response (18). In Alzheimer’s disease, neuroinflammation is a pathological feature exacerbated by the accumulation of amyloid-beta plaques through the activation of inflammatory proteins including IL-1, IL-6, and TNF-α. Interestingly, magnesium supplementation has been shown to reduce the levels of these proinflammatory cytokines and increase the levels of anti-inflammatory mediators in the hippocampus of a rat model of Alzheimer’s disease, suggesting modulation of an inflammatory responses (19). However, due to the complexity of the immune system in the brain, with the involvement of microglia, astrocytes, and various cytokines and chemokines, dampening inflammation alone might not be sufficient. Chronic neuroinflammation results in an adverse cascade of events, causing neuronal damage, disrupting synaptic functionality, and leading to cognitive impairment. When this inflammatory response is sustained, it results in the overproduction of proinflammatory cytokines. This hyperreactive state disrupts the delicate balance of synaptic plasticity (the ability of synapses to strengthen or weaken over time) thereby diminishing key cognitive functions like memory retention and learning (20).

Furthermore, prolonged inflammation triggers oxidative stress, wherein excess free radicals lead to neurotoxicity and cellular damage (9). This accelerates the progression of neurodegenerative processes, as observed in diseases such as Alzheimer’s disease and Parkinson’s disease, which are characterized by the accumulation of disease-specific proteins in the brain, amyloid-beta and alpha-synuclein, respectively (21). Additionally, inflammation-induced oxidative stress and resultant neuronal damage have been identified as significant contributors to cognitive decline following traumatic brain injury. These findings illustrate the detrimental link between chronic neuroinflammation and cognitive decline.

## Magnesium deficiency syndromes

Hypomagnesemia (typically below 0.61 mmol/L) can cause a wide range of disorders and has significant neurological consequences. The causes of hypomagnesemia can be related to gastrointestinal disorders, including chronic diarrhea, malabsorption syndromes (e.g., celiac disease, inflammatory bowel disease), chronic pancreatitis, and excessive vomiting. Similarly, renal disorders, including tubular dysfunction, diabetic nephropathy (leading to increased urinary magnesium loss), and the use of certain medications (e.g., diuretics, proton pump inhibitors, and some antibiotics), can cause hypomagnesemia. Alcoholism, severe burns, chronic stress, hyperaldosteronism, and prolonged parenteral fluid administration without magnesium supplementation can also lead to hypomagnesemia. Magnesium plays a key role in neural function, and its inadequacy can lead to various neurological symptoms and complications, including neuromuscular hyperexcitability, muscle twitches and cramps, tremors, and seizures. Of clinical importance, the severity of neurological symptoms often correlates with the severity of magnesium deficiency. Patients with mild hypomagnesia (below 0.61 mmol/L) may cause subtle symptoms, while severe hypomagnesia (below 0.49 mmol/L) can lead to more pronounced neurological manifestations. Severe magnesium deficiency syndromes can be associated with cognitive and mood disturbances, headaches, migraines, and neuropathy (numbness and tingling sensations, particularly in the extremities). The long-term complications of severe magnesium deficiency have also been linked to nystagmus (involuntary eye movements) and neurodegenerative diseases, possibly mediated by neuroinflammation. It is essential to maintain an optimal balance of magnesium, along with other minerals and vitamins, throughout life to support normal physiologic functions, including neurological health (22–25).

## Role of magnesium in neuroinflammation

In the nervous system, magnesium is essential for maintaining neuronal ion homeostasis, modulating synaptic plasticity, and regulating neurotransmitter release (26). Kang et al. highlighted the integral role of this mineral in managing the activity of N-methyl-D-aspartate (NMDA) receptors (27). Their findings emphasize the significance of this interaction in maintaining the balance of glutamate, an excitatory neurotransmitter. If left unchecked, glutamate can potentially tip the scale toward inflammation. Kramer et al. suggested the aftereffects of magnesium deficiency (28). According to their findings, insufficient magnesium can trigger an increase in substance P, a neuropeptide that propagates inflammatory pain (28).

Other researchers have highlighted the complex interplay between magnesium and calcium within neurons (9). By restraining calcium influx into neurons, magnesium helps prevent events that could otherwise lead to intensified inflammation and neuronal injury. Whereas low levels of this mineral are associated with chronic inflammation, restoring magnesium balance has been shown to potentially counteract this condition (29). Apart from managing neurotransmitter activity, magnesium has been found to play a crucial role in modulating immune responses, particularly by interacting with nuclear factor kappa-light-chain-enhancer of activated B cells (NF-κB) (19). This research provides compelling evidence of the role of magnesium as an NF-κB inhibitor, a transcription factor that regulates the expression of pro-inflammatory cytokines, including TNF-α and IL-6 (19). By inhibiting NF-κB activation, magnesium can dampen the resultant proinflammatory gene expression, thereby reducing the overall inflammatory response within the brain (19). A meta-analysis by Veronese et al. revealed magnesium’s anti-inflammatory effects, marked by reductions in plasma fibrinogen and other markers, such as tartrate-resistant acid phosphatase type 5 (TRACP 5) and tumor necrosis factor-ligand superfamily member 13B (TNFSF13B) (30). Additionally, it was also noticed that ST2 protein and IL-1 levels went down. However, the study revealed no significant changes in IL-6 or total antioxidant capacity levels, indicating a selective impact of magnesium on various inflammatory markers (30). Of clinical significance, measuring circulating ionized magnesium appears to be a more accurate indicator of magnesium supplement bioavailability compared to assessing total magnesium levels in plasma (31). Although the role of magnesium in regulating neurotransmission and immune responses is well established, it also plays a crucial role in maintaining brain health by acting as an antioxidant. Research findings suggest that magnesium may contribute to neutralizing ROS to delay the progression of neurodegenerative disorders (32).

Although magnesium is not considered a component of the antioxidant defense system, research indicates that magnesium deficiency may increase oxidative stress markers. These markers encompass oxidative modification products of lipids, proteins, and DNA. Furthermore, a significant association was observed between magnesium deficiency and weakened antioxidant defense mechanisms. This relationship between magnesium deficiency and oxidative stress involves multifaceted mechanisms at both the systemic and cellular levels, including inflammation, endothelial dysfunction, mitochondrial dysfunction, and excessive fatty acid production (32). The studies suggest that magnesium may possess inherent antioxidant properties, although not as a conventional antioxidant molecule such as vitamin C or vitamin E. One notable mechanism highlighted is magnesium’s role in stabilizing the critical antioxidant enzyme superoxide dismutase (SOD) (32). SOD substantially mitigates oxidative damage by converting harmful superoxide radicals into less reactive molecular species. This stabilization of SOD by magnesium provides a unique and essential link between magnesium and the antioxidant defense system (32).

## Magnesium and neuroprotection

Neuroprotective agents are substances that can potentially preserve neuronal structure and function. These substances help prevent or slow the progression of neurodegenerative diseases, such as Alzheimer’s and Parkinson’s disease. These agents work through various mechanisms, including reducing neuroinflammation, shielding against oxidative stress, and modulating neurotransmission (33).

**Figure 2:**
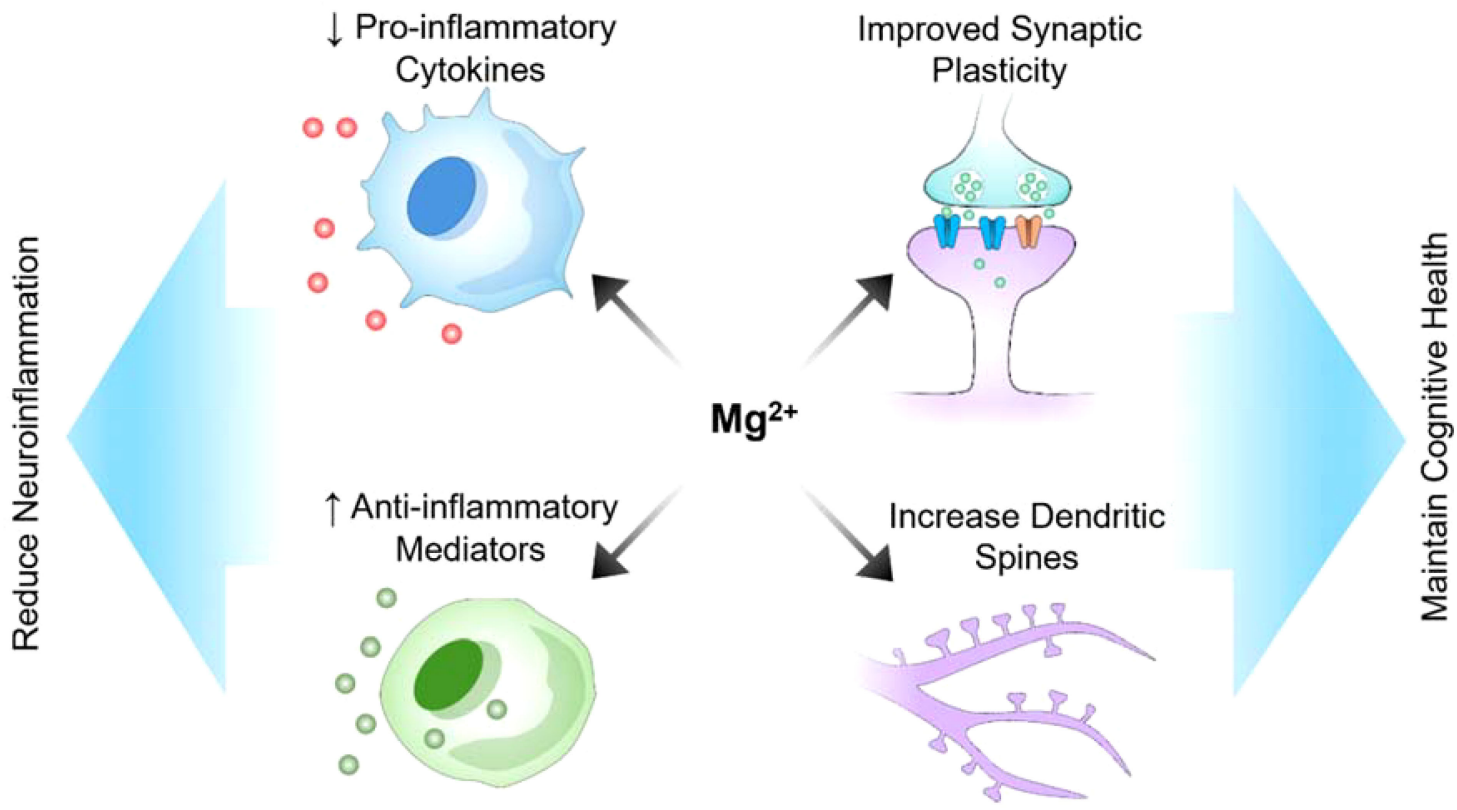
Potential role of magnesium in reducing neuroinflammation and maintaining cognitive health.

Many preclinical and clinical studies have suggested the potential of magnesium as a neuroprotective agent (**Figure 2**). Magnesium is present both intracellularly and extracellularly, with its intracellular presence in compartments such as the nuclei, mitochondria, and endoplasmic reticulum being crucial for central nervous system functions, including synaptic connectivity (34). Intracellular magnesium can modify synaptic properties, influencing various neuronal processes. For instance, recent research by Liu’s group reported that presynaptic intracellular magnesium plays a crucial role in mediating the transition between two synaptic configurations: one involved in information encoding and learning, and the other in information storage and memorization (35). Research has demonstrated that magnesium can enhance cognitive function and synaptic plasticity in animal models of Alzheimer’s disease, offering optimism for addressing cognitive decline (10). Additionally, a study conducted on a rat model of Alzheimer’s disease demonstrated that magnesium sulfate supplementation improved cognitive function, synaptic plasticity, and dendritic spine morphology (10). Moreover, intracellular magnesium levels have been shown to correlate with Parkinson’s disease activity. In 1-methyl-4-phenylpyridinium (MPP+) model of Parkinson’s disease, the application of MPP+ induced an increase in intracellular magnesium concentration, which inhibited cellular ROS production, maintained ATP generation, and preserved cell viability, thereby protecting neurons from MPP+ toxicity (36). In demyelination rat models, a mutation in the mitochondrial magnesium uptake gene disrupted magnesium homeostasis in oligodendrocytes, affecting ATP production and leading to axonal demyelination (37). Besides supporting myelin formation, intracellular magnesium also enhanced oligodendrocytes’ tolerance against cellular stress, increasing resistance to a hypoxic-ischemic injury (38). Although preclinical studies suggest that magnesium has potential neuroprotective effects, translating these findings to humans presents numerous challenges. Differences in metabolism, blood−brain barrier permeability, and magnesium bioavailability between humans and animal models may affect its efficacy in clinical settings. Additionally, the optimal dosage, duration of treatment, and form of magnesium (e.g., magnesium sulfate, magnesium citrate, etc.) that are both effective and safe for humans require rigorous clinical trials. A gap exists between demonstrating neuroprotection under controlled laboratory conditions and achieving measurable, meaningful outcomes in diverse human populations with varying stages of neurological conditions. Supplementation with magnesium sulfate increased brain magnesium contents and attenuated memory deficits induced by intracerebroventricular administration of streptozocin (ICV-STZ). Furthermore, magnesium reduces tau hyperphosphorylation, a hallmark of Alzheimer’s disease, and modulates the PI3K/Akt signaling pathway (10). Additionally, magnesium supplementation has been associated with improved neurological outcomes in models of acute brain injury, demonstrating its relevance in central nervous system injuries (39). Moreover, in an experimental setting involving a rat model of sciatic nerve injury, a diet rich in magnesium was found to stimulate neurological function recovery and enhance nerve regeneration, revealing its potential in the treatment of peripheral nerve disorders (39). The neuroprotective effects of magnesium are believed to stem from its capacity to regulate neuronal calcium homeostasis, thus reducing excitotoxicity, and its ability to modulate neuroinflammatory processes (10). The mechanisms by which magnesium exerts its effects (e.g., calcium homeostasis regulation, reduction in excitotoxicity, anti-inflammatory actions) suggest that its neuroprotective properties could be applicable to a wide range of neurological conditions. However, this also raises questions about specificity and targeted therapy. For instance, although reducing tau hyperphosphorylation is promising for treating Alzheimer’s disease, it is unclear how these mechanisms interact in the presence of other neurodegenerative disorders or comorbidities. The multifunctional nature of magnesium might mean that its efficacy could vary greatly depending on the specific pathological context. Additionally, magnesium appears to influence nitric oxide production; nitric oxide is a molecule critical for regulating cerebral blood flow and neuronal damage.

Between 2002 and 2008, several randomized clinical trials explored the potential of magnesium sulfate for neuroprotection in preterm births and its effects on cerebral palsy (40). Although these studies did not consistently achieve statistical significance for their primary outcomes, they indicated that magnesium sulfate exposure significantly reduced the likelihood of cerebral palsy in preterm infants. A similar clinical study by Temkin et al., 2007 involving 499 participants aimed to test whether magnesium treatment favorably affects outcomes in head-injured patients (41). The results show that participants who were randomly assigned to the lower dose magnesium group performed significantly worse than those in the placebo group. Therefore, there was greater mortality with the magnesium dose than with the placebo. These findings highlight a discrepancy between preclinical expectations and clinical observations, suggesting that the magnesium infusions given to patients within 8 hours of traumatic brain injury did not have a neuroprotective effect on traumatic brain injury (41). However, other studies have claimed that intravenous magnesium infusion and hyperbaric oxygen therapy could reduce the clinical symptoms of brain injury (42–44). Therefore, additional pre-clinical and clinical research is needed to provide stronger scientific validation.

Another study investigated the combined effects of magnesium supplementation and treadmill exercise on memory deficits in aged rats (45); combined approach led to improved memory function in the aged rats. In the context of central nervous system injury, a comprehensive review highlighted the significant decrease in blood and brain (free) magnesium concentrations following both direct and indirect neurotrauma (46). A decrease in magnesium was associated with neurological deficits and oxidative stress, emphasizing the importance of magnesium homeostasis in central nervous system injury. The administration of magnesium salts, such as magnesium sulfate and magnesium chloride, increased brain (free) magnesium concentrations and improved functional outcomes (46).

## The cognitive lifeline: magnesium supplementation

Research has demonstrated that magnesium supplementation can effectively increase extracellular magnesium levels, particularly in the serum, which may help inhibit the aggregation of calciprotein particles and reduce vascular calcifications, helping manage conditions such as chronic kidney disease (47). However, the effects on intracellular magnesium levels are more complex and require a long-term, consistent approach to supplementation. This slow adjustment is necessary because of the body’s regulatory mechanisms, which ensure that cellular functions remain stable and effective.

In neurological disorders such as Alzheimer’s disease and Parkinson’s disease, the neurodegenerative process has occurred for many years, potentially reducing the responsiveness of these disorders to the benefits of magnesium. Magnesium impacts calcium regulation and neurotransmitter functions, which are implicated in the pathophysiology of these diseases. In Parkinson’s disease, abnormal magnesium levels are linked to transporter dysfunctions, suggesting that supplementation could stabilize these transport mechanisms and potentially slow disease progression (48).

Conversely, in acute neurological conditions such as stroke or traumatic brain injury, rapid onset and progression do not allow magnesium levels to be corrected in a timeframe that influences immediate outcomes. In these cases, emergency treatments focus on restoring blood flow or reducing inflammation rather than correcting metabolic imbalances. The slow cellular uptake and regulatory effects of magnesium are less practical here because the therapeutic window is very narrow, and the rapid physiological changes postinjury require immediate interventions that go beyond magnesium supplementation. Therefore, while chronic neurological disorders could benefit from sustained magnesium research owing to their slow progression, acute disorders would receive minimal benefit from such research. This is due to need for immediate and aggressive treatment in acute conditions, where the timing and rapid action are critical.

Magnesium supplementation varies significantly in form and administration, each tailored for specific clinical scenarios. Oral magnesium, available in forms such as oxide, citrate, and glycinate, is commonly used for long-term management of conditions such as cardiovascular health and migraine prophylaxis. These forms are preferred for their high bioavailability and ease of administration, making them ideal for ongoing, nonemergency supplementation. Conversely, intravenous magnesium, primarily known as magnesium sulfate, is used in emergency settings where rapid correction of magnesium levels is critical. This form is used in acute medical conditions such as severe asthma, eclampsia, or life-2threatening arrhythmias. Direct administration into the bloodstream provides an immediate therapeutic effect, which is crucial in life-saving interventions. Topical magnesium, often in the form of oils or gels, is used for local applications such as muscle soreness and cramps. While it offers the advantages of bypassing the gastrointestinal system and avoiding some side effects associated with oral forms, its systemic absorption and overall efficacy are less documented.

The relationship between magnesium intake and cognitive function is a promising research area. A study from the National Health and Nutrition Examination Survey (NHANES) 2011 to 2014 investigated the associations of vitamin D status and magnesium with cognitive status in older adults (49). The study found that higher serum 25-hydroxyvitamin D [25(OH)D] levels, linked with magnesium metabolism, were associated with reduced risk of declining cognitive function. Specifically, an inverse association of higher serum 25(OH)D levels with cognitive function was observed primarily among participants with a daily total magnesium intake of <254 mg or ≤375 mg. Essential roles of magnesium in the activation of vitamin D has been explained in various research publications (49–53). The associations between serum 25(OH)D and risk of mortality may be modified by the intake level of magnesium (49). Nevertheless, some studies reportered that there were no clear associations for cognitive function with overall magnesium intake (54). Although not directly focused on magnesium, research has highlighted the potential cognitive benefits of other dietary components. For instance, a study conducted in Qatar revealed that habitual consumption of nuts (almonds, cashews, Brazil nuts, and walnuts), which are rich in magnesium, is positively associated with cognitive function, especially among older adults (55).

Furthermore, a multicenter study of hemodialysis patients revealed a U-shaped association between serum magnesium levels and mild cognitive impairment. Both lower and higher serum magnesium levels were observed to increase the risk of mild cognitive impairment in this specific population. The optimal range of magnesium levels for the lowest risk of mild cognitive impairment was identified as 1.12–1.24 mmol/L (56). This discrepancy suggests that while the current reference range for serum magnesium (0.75–0.95 mmol/L) may be adequate for typical physiological functions, higher levels would be necessary for optimal cognitive health. This indicates that standard ranges might not fully address the specific needs of the brain and neurological health. Therefore, maintaining serum magnesium levels at the higher end of the range could provide potential neuroprotective benefits. The empirical data from specialized populations like hemodialysis patients delineate magnesium’s potential as a cognitive lifeline. The observed associations between magnesium levels and cognitive outcomes highlight the significance of this mineral and raise questions about optimal intake levels for cognitive preservation.

Magnesium glycinate, known for its high bioavailability, ensures that magnesium is efficiently absorbed into the bloodstream and, consequently, available to the body and brain (57). Although direct studies on the impact of magnesium glycinate on cognitive function are limited, its role in enhancing sleep quality and reducing anxiety could indirectly support cognitive health by promoting restorative sleep and lowering stress levels, both of which are beneficial for cognitive performance and neuroprotection (58). Magnesium L-threonate has been specifically studied for its unique ability to increase magnesium concentrations in the brain, thus directly influencing cognitive functions. Rats supplemented with magnesium L-threonate showed a significant increase in synaptic density in regions of the brain associated with memory and learning, translating to a 15% improvement in maze navigation tasks compared to controls. This study demonstrated that this form of magnesium could reverse certain aspects of brain aging and improve synaptic density, suggesting that magnesium has promising implications for delaying and treating cognitive decline associated with aging and neurodegenerative diseases (59).

The existing body of research underscores the need for more rigorous, long-term clinical trials to provide conclusive evidence. A study by Nosheny et al. emphasized the role of dyadic cognitive reports and subjective cognitive decline in early Alzheimer’s disease research and trials (60). Although this study did not focus on magnesium directly, it highlighted the importance of long-term monitoring and the complexities in data interpretation, suggesting that similar rigorous methodologies should be applied to studies on magnesium. Furthermore, research by Planche et al. on brain atrophy subtypes during aging indicated that certain atrophy patterns might predict long-term cognitive decline and future Alzheimer’s disease (61).

## Conclusion

The role of magnesium in cognitive health and neuroprotection is both compelling and complex, a testament to the sophisticated nature of the nervous system and its interplay with essential nutrients. Research has revealed that magnesium is a critical player in maintaining and regulating neurobiological behaviors. In fact, its ability to mediate inflammatory signaling pathways and inhibit the activation of NF-κB provides a basis for its potent anti-inflammatory effects. By reducing oxidative burden and inflammation (two phenomena significantly contributing to cognitive decline), magnesium helps to preserve neuronal integrity. Epidemiological and clinical research consistently stresses the importance of adequate magnesium levels for improving cognitive health. Studies have shown a direct correlation between magnesium intake and cognitive function in healthy individuals. Although existing studies have laid a substantial foundation, they also highlight the need for further in-depth research, including more comprehensive, long-term clinical trials to determine the therapeutic potency of magnesium in improving cognitive health to provide safe and compassionate patient care (62), to reduce the burden of neurodegenerative diseases.
